# Evaluation of the effects of pemafibrate on metabolic dysfunction‐associated steatotic liver disease with hypertriglyceridemia using magnetic resonance elastography combined with fibrosis‐4 index and the magnetic resonance imaging‐aspartate aminotransferase score

**DOI:** 10.1002/jgh3.13012

**Published:** 2023-12-11

**Authors:** Takeshi Ichikawa, Haruki Oba, Mai Owada, Kazuki Watanabe, Tsubasa Yoshimura, Ayako Fuchigami, Atsushi Nakamura

**Affiliations:** ^1^ Department of Gastroenterology and Hepatology Nippon Koukan Hospital Kawasaki Japan

**Keywords:** hyperglyceridemia, magnetic resonance elastography combined with fibrosis‐4 index, magnetic resonance imaging‐aspartate aminotransferase score, metabolic dysfunction‐associated steatotic liver disease, pemafibrate

## Abstract

**Background and Aim:**

In this retrospective study, we evaluated the effects of pemafibrate treatment in patients with metabolic dysfunction‐associated steatotic liver disease (MASLD) and hypertriglyceridemia using non‐invasive stiffness‐based models, including magnetic resonance elastography (MRE) combined with the fibrosis‐4 (FIB‐4) (MEFIB) index and the magnetic resonance imaging (MRI)‐aspartate aminotransferase (AST) (MAST) score.

**Methods:**

In total, 179 patients with MASLD treated with pemafibrate were enrolled. We evaluated the effects of 48‐week pemafibrate treatment using the MEFIB index, which classifies patients based on the combination of liver stiffness measurement (LSM) on MRE and FIB‐4 and the MAST score, which is calculated based on LSM on MRE, MRI‐proton density fat fraction (MRI‐PDFF), and AST levels.

**Results:**

Pemafibrate treatment led to significant reduction in AST, alanine aminotransferase (ALT), and gamma‐glutamyl transferase (GGT) (*P* = 0.011, <0.001, and <0.001, respectively) and significant improvements in triglyceride and high‐density lipoprotein cholesterol levels (*P* < 0.001 and <0.001, respectively). The MRI‐PDFF values were not significantly altered. However, a significant decrease in LSM on MRE was detected (*P* = 0.003). Evaluation of fibrosis using the MEFIB index and MAST score demonstrated significant improvement (*P* = 0.004 and <0.001, respectively). Changes in the MAST score showed positive correlation with changes in ALT and GGT levels (*r* = 0.821, *P* < 0.001, and *r* = 0.808, *P* < 0.001, respectively). Additionally, ALT and GGT levels at baseline were significantly associated with improvements in the MAST score (*P* < 0.001 and <0.001, respectively).

**Conclusion:**

Pemafibrate led to improvements in the MEFIB index and MAST score, as well as liver function. It is a promising therapeutic agent for patients with MASLD and hypertriglyceridemia with the potential to reduce liver‐related events.

## Graphical abstract

This retrospective study evaluated the effects of pemafibrate treatment in patients with MASLD and hypertriglyceridemia using MR elastography combined with FIB‐4 (MEFIB) index and MRI‐AST (MAST) score. Pemafibrate treatment led to significant reduction in AST, ALT, and GGT. Evaluation of fibrosis using MEFIB index and MAST score demonstrated significant improvement.

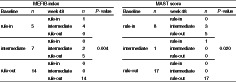

## Introduction

The global incidence of nonalcoholic fatty liver disease (NAFLD) is on the rise. NAFLD affects 25% of the general population worldwide.^1^ It is associated with obesity, type 2 diabetes, dyslipidemia, and hypertension and can progress to nonalcoholic steatohepatitis (NASH), which increases the risk of developing cirrhosis and hepatocellular carcinoma. Recently, NASH has become one of the leading indications for liver transplantation in the United States and Europe.^2^, ^3^

Although the guidelines for the management of NAFLD/NASH recommend lifestyle interventions with diet modification and exercise for weight management,^4^, ^5^, ^6^ the response to such interventions remains limited for many patients. A few effective pharmacotherapies for NAFLD/NASH have been established.^4^, ^5^, ^6^ However, none of them has gained approval for the treatment yet.^7^

Peroxisome proliferation‐activated receptors (PPARs) are members of the nuclear receptors superfamily of ligand‐activated transcription factors^8^ containing three isotypes (α, δ, γ) that form a subfamily.^9^ PPARα has key roles in the regulation of fatty‐acid transport as well as peroxisomal and mitochondrial β‐oxidation in the liver.^10^ It is associated with the transcription of genes involved in reducing serum triglyceride (TG) and increasing high‐density lipoprotein cholesterol (HDL‐C) and regulation of lipid and lipoprotein metabolism.^11^

Pemafibrate is a selective PPARα modulator, which was designed to exhibit a higher PPARα agonistic activity and selectivity than existing PPARα agonists such as fibrates.^12^ It was approved for the treatment of hyperlipidemia in July 2017 and has been marketed in Japan since June 2018.^13^ Pemafibrate maximizes fibrate's beneficial effects and minimizes its adverse effects. Although fibrates have demonstrated worsening of liver and kidney function test results, pemafibrate significantly reduces the levels of alanine aminotransferase (ALT) and gamma‐glutamyl transpeptidase (GGT) without increasing serum creatinine or decreasing estimated glomerular filtration rate (eGFR) in patients with NAFLD.^14^, ^15^, ^16^, ^17^, ^18^, ^19^

Liver biopsy has limitations due to potential sampling errors and intra‐ and inter‐observer variability. Moreover, it is also an invasive procedure with adverse effects, such as pain, risk of infection, bleeding, perforation, and, albeit rarely, even death. Noninvasive identification of candidates with high positive predictive value (PPV) is a major unmet need for pharmacologic treatment.^20^, ^21^ FibroScan‐aspartate aminotransferase (FAST) score has emerged as a useful method for noninvasively identifying patients with NASH who have significant activity and advanced fibrosis, through a combination of liver stiffness measurement (LSM) via vibration‐controlled transient elastography (VCTE), controlled attenuation parameter (CAP), and aspartate aminotransferase (AST).^22^ Magnetic resonance imaging‐proton density fat fraction (MRI‐PDFF) has superior accuracy to VCTE‐based CAP in identifying all grades of steatosis in patients with NAFLD.^23^, ^24^ Previous studies have also demonstrated that magnetic resonance elastography (MRE) is more accurate than VCTE in detecting liver fibrosis in patients with NAFLD.^24^

LSM on MRE combined with fibrosis‐4 (FIB‐4) (the MEFIB index) has proven its utility in identifying candidates (≥stage 2 fibrosis) for pharmacologic therapy in patients with NAFLD.^21^ Additionally, the MRI‐AST (MAST) score, which was developed based on the combination of LSM on MRE, MRI‐PDFF, and AST levels, has emerged as a potent tool to identify patients with NAFLD who are at a higher risk of disease progression to fibrosis and cirrhosis.^25^ These MRI‐based, noninvasive assessments outperform previous noninvasive scores, such as the FIB‐4 index, the NAFLD fibrosis score, and the FAST score, in identifying patients with fibrotic NASH (Fibro‐NASH).^21^, ^25^, ^26^, ^27^ Both the MEFIB index and the MAST score have demonstrated correlation with histological measures of fibrosis,^28^, ^29^ with the latter seemingly a useful marker of treatment response.^28^

Recently, the diagnostic criteria and definition for NAFLD have been revised to metabolic dysfunction‐associated steatotic liver disease (MASLD). The new nomenclature is nonstigmatizing and can improve awareness and patient identification.^30^, ^31^, ^32^

In this study, we have evaluated the effects of pemafibrate in patients with MASLD and hypertriglyceridemia, focusing on liver enzymes and the utilization of MRI‐based noninvasive tests.

## Methods

### Patients

This retrospective single‐center cohort study included 179 patients with MASLD and hypertriglyceridemia who were treated with pemafibrate at Nippon Koukan Hospital between March 2019 and November 2021. All patients were diagnosed with fatty liver disease using abdominal ultrasonography (US). Fatty changes were noted when the patients exhibited increased hepatic echogenicity, increased liver–kidney contrast, deep US attenuation in the liver, and/or poor visualization of the hepatic vein. Hypertriglyceridemia was diagnosed based on an elevated level of fasting TG (≥150 mg/dL) or non‐fasting TG (≥175 mg/dL). Patients with excessive alcohol consumption (≥30 g/day for men and ≥20 g/day for women), chronic hepatitis due to other causes such as hepatitis B and hepatitis C virus, autoimmune hepatitis, primary biliary cholangitis, decompensated cirrhosis, and hepatocellular carcinoma were excluded from the analysis. All patients met the criteria for MASLD.^30^, ^31^, ^32^ Patients were prescribed pemafibrate (oral, 0.1 mg, twice a day) and instructed to visit the outpatient clinic every 4–12 weeks. The Common Terminology Criteria for Adverse Events version 5.0 was used to evaluate the adverse events associated with pemafibrate. The Ethical Committee of Nippon Koukan Hospital approved this retrospective study and waived the requirement for obtaining informed consent from the patients.

### Laboratory and clinical parameters

The patients underwent biochemical examination to assess their lipid profile, liver function, and renal function every 4–12 weeks. Among the patients, a subgroup of 26 patients underwent an investigation of the effect of LSM on MRE and liver fat content on MRI‐PDFF at baseline and at week 48. LSM on MRE and the FIB‐4 index were used to evaluate the MEFIB index. The MEFIB index employed the rule‐out (MRE < 3.3 kPa and FIB‐4 index <1.6) and rule‐in (MRE ≥ 3.3 kPa and FIB‐4 index ≥1.6) criteria for stratification, based on a previous study.^21^ The MAST score was calculated as follows: MAST = −12.17 + 7.07 log MRE + 0.037 PDFF + 3.55 log AST.^25^ MAST scores of <0.165 and ≥0.242 served as the rule‐out and rule‐in criteria, respectively. Furthermore, changes in LSM on MRE, liver fat content on MRI‐PDFF, MEFIB index, and MAST score from baseline to week 48 were assessed.

### Statistical analyses

Continuous data for biochemical examinations are expressed as mean ± standard deviation, and categorical data are presented as numbers (percentage). The paired Wilcoxon test and chi‐square test were used to analyze the differences in each parameter between baseline and week 48. The correlation between changes in the MAST score at week 48 and baseline factors as well as changes in these factors at week 48 were assessed using Spearman's rank correlation coefficient. *P*‐values of <0.05 were considered to indicate statistical significance. All statistical analyses were performed using SAS 8 software (SAS Institute).

## Results

### Baseline characteristics of the study patients

Table 1 summarizes the patients' demographic profiles. The mean age of the patients was 59.9 years, and 126 (70.4%) of them were men. Their mean body mass index (BMI) was 27.7 kg/m^2^. Type 2 diabetes, hypertension, and chronic kidney disease were diagnosed in 77 (43.0%), 123 (68.7%), and 45 patients (25.1%), respectively. Antidiabetics, namely sodium‐glucose cotransporter 2 inhibitor, glucagon‐like peptide‐1 agonist, and thiazolidinedione, were prescribed to 18 (10.1%), 1 (0.6%), and 1 (0.6%) patients, respectively. Antilipidemics, statins, ezetimibe, eicosapentaenoic acid (EPA), EPA/docosahexaenoic acid preparation, and vitamin E were prescribed to 62 (34.6%), 6 (3.4%), 6 (3.4%), 4 (2.2%), and 4 (2.2%) patients, respectively.

**Table 1:** Patients characteristics (*n* = 179)

Age (years)	59.9 ± 13.6
Male, *n* (%)	126 (70.4)
Body weight (kg)	75.1 ± 14.6
BMI (kg/m^2^)	27.7 ± 4.4
BMI (kg/m^2^) > 25	127 (70.9)
Comorbidities, *n* (%)	
Type 2 diabetes	77 (43.0)
Hypertension	123 (68.7)
Chronic kidney disease	45 (25.1)
Concomitant medications, *n* (%)	
Antidiabetics	
SGLT2 inhibitor	18 (10.1)
GLP‐1 agonist	1 (0.6)
Thiazolidinedione	1 (0.6)
Antilipidemics	
Statin	62 (34.6)
Ezetimibe	6 (3.4)
EPA	6 (3.4)
EPA/DHA preparation	4 (2.2)
Other medications	
Vitamin E	4 (2.2)

Data are presented as mean ± SD.

BMI, body mass index; DHA, docosahexaenoic acid; EPA, eicosapentaenoic acid; GLP‐1, glucagon‐like peptide‐1; SGLT2, sodium‐glucose cotransporter 2.

### Changes in laboratory and clinical data

Table 2 presents the changes in each factor after 48 weeks of pemafibrate treatment. During pemafibrate treatment, total bilirubin, AST, ALT, and GGT levels significantly decreased (*P* < 0.001, 0.011, <0.001, and <0.001, respectively), whereas serum albumin levels and platelet counts significantly increased (*P* < 0.001 and <0.001, respectively). Additionally, serum TG, HDL‐C, and non‐HDL‐C levels significantly improved (*P* < 0.001, <0.001, and <0.001, respectively). Notably, body weight and eGFR were not significantly altered during pemafibrate treatment. There was no clinically relevant change also in hemoglobin A1c and creatine kinase. Two patients discontinued pemafibrate treatment owing to adverse events (pruritus and rash).

**Table 2:** Comparison of values at baseline and week 48

	Baseline	Week 48	*P‐*value
Body weight (kg)	75.1 ± 14.5	76.3 ± 15.2	0.094
Total bilirubin (mg/dL)	0.80 ± 0.33	0.68 ± 0.25	<0.001
AST (U/L)	31.6 ± 23.1	28.3 ± 14.8	0.011
ALT (U/L)	42.5 ± 40.3	32.0 ± 23.3	<0.001
GGT (U/L)	59.6 ± 61.1	38.8 ± 35.7	<0.001
Albumin (g/dL)	4.34 ± 0.33	4.47 ± 0.52	<0.001
eGFR (mL/min/1.73 m^2^)	69.1 ± 23.2	68.2 ± 24.9	0.698
TG (mg/dL)	376.7 ± 346.0	196.9 ± 137.1	<0.001
LDL‐C (mg/dL)	116.6 ± 32.2	122.0 ± 32.4	0.139
HDL‐C (mg/dL)	45.4 ± 11.3	48.3 ± 11.6	<0.001
Non‐HDL‐C (mg/dL)	170.1 ± 47.1	150.3 ± 42.3	<0.001
HbA1c (%)	6.4 ± 1.0	6.5 ± 0.9	0.251
CK (U/L)	126.8 ± 87.0	126.9 ± 81.3	0.970
Platelets (10^4^/μL)	24.6 ± 5.9	26.1 ± 6.0	<0.001
FIB‐4 index	1.31 ± 0.64	1.32 ± 0.62	0.715

Data are presented as mean ± SD.

ALT, alanine aminotransferase; AST, aspartate aminotransferase; CK, creatine kinase; eGFR, estimated glomerular filtration rate; GGT, gamma‐glutamyl transferase; HbA1c, hemoglobin A1c; HDL‐C, high‐density lipoprotein cholesterol; LDL‐C, low‐density lipoprotein cholesterol; TG, triglyceride.

### Changes in the evaluation of significant fibrosis using the MEFIB index and MAST score at week 48

Table 3 presents the changes in the diagnosis based on the rule‐in and rule‐out criteria as determined using the MEFIB index and MAST score. Among five patients diagnosed using the rule‐in criteria based on MEFIB index at baseline, four showed improvement to the intermediate level following pemafibrate treatment. Among seven patients initially classified as intermediate based on the MEFIB index, five patients showed improvement to meet the rule‐out criteria following treatment. These findings indicated that pemafibrate treatment significantly improved the evaluation of significant fibrosis based on the MEFIB index (*P* = 0.004). Among the eight patients diagnosed using the rule‐in criteria based on the MAST score, five were reclassified according to the rule‐out criteria and three were classified as intermediate following treatment. These findings indicate that the evaluation of significant fibrosis using the MAST score demonstrates improvement following pemafibrate treatment (*P* = 0.020).

**Table 3:** Changes in the evaluation of significant fibrosis using the MEFIB index and MAST score at week 48

MEFIB index					MAST score				
Baseline	*n*	Week 48	*n*	*P‐*value	Baseline	*n*	Week 48	*n*	*P*‐value
Rule‐in	5	Rule‐in	1	0.004	Rule‐in	8	Rule‐in	0	0.020
		Intermediate	4				Intermediate	3	
		Rule‐out	0				Rule‐out	5	
intermediate	7	Rule‐in	0		Intermediate	1	Rule‐in	1	
		Intermediate	2				Intermediate	0	
		Rule‐out	5				Rule‐out	0	
Rule‐out	14	Rule‐in	0		Rule‐out	17	Rule‐in	0	
		Intermediate	0				Intermediate	0	
		Rule‐out	14				Rule‐out	17	

MAST, magnetic resonance imaging‐aspartate aminotransferase; MEFIB, magnetic resonance elastography combined with FIB‐4.

### Changes in MRE, MRI‐PDFF, and MAST score

**Figure 1:**
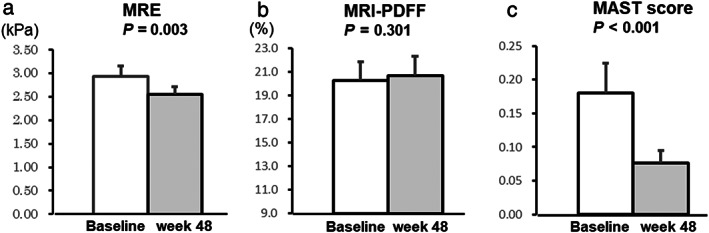
Evaluation of the changes in (a) liver stiffness using magnetic resonance elastography (MRE), (b) liver fat content using magnetic resonance imaging‐proton density fat fraction (MRI‐PDFF), and (c) magnetic resonance imaging‐aspartate aminotransferase (MAST) score between baseline and week 48 of pemafibrate treatment. Data are expressed as mean with standard error of the mean.

Figure 1 shows the changes in each parameter at baseline and after 48 weeks of treatment. The LSM on MRE significantly decreased from 2.93 to 2.55 (*P* = 0.003). Additionally, the MAST score significantly decreased from 0.18 to 0.08 (*P* < 0.001). In contrast, the liver fat content measured using MRI‐PDFF did not change significantly from baseline to week 48.

### Factors at baseline associated with changes in MAST score

Table 4 presents the association between changes in the MAST score and baseline factors. The levels of AST, ALT, and GGT at baseline showed negative correlations with the changes in the MAST score (*r* = −0.849, *P* < 0.001; *r* = −0.793, *P* < 0.001; and *r* = −0.766, *P* < 0.001, respectively). Additionally, the FIB‐4 index, LSM on MRE, and the MAST score at baseline showed correlations with the changes in the MAST score (*r* = −0.534, *P* = 0.005; *r* = −0.567, *P* = 0.003; and *r* = −0.911, *P* < 0.01, respectively).

**Table 4:** Association between changes in magnetic resonance imaging‐aspartate aminotransferase (MAST) score and baseline factors

At baseline	Change in MAST score	
	Correlation coefficient	*P*‐value
Age (years)	0.269	0.183
Body weight (kg)	−0.336	0.094
Total bilirubin (mg/dL)	0.059	0.777
AST (U/L)	−0.849	<0.001
ALT (U/L)	−0.793	<0.001
GGT (U/L)	−0.766	<0.001
eGFR (mL/min/1.73 m^2^)	−0.237	0.243
TG (mg/dL)	0.010	0.963
LDL‐C (mg/dL)	−0.300	0.137
HDL‐C (mg/dL)	0.018	0.928
Non‐HDL‐C (mg/dL)	−0.376	0.058
Platelets (10^4^/μL)	0.167	0.414
FIB‐4 index	−0.534	0.005
MRE (kPa)	−0.567	0.003
MRI‐PDFF (%)	−0.271	0.180
MAST score	−0.911	<0.001

ALT, alanine aminotransferase; AST, aspartate aminotransferase; eGFR, estimated glomerular filtration rate; GGT, gamma‐glutamyl transferase; HDL‐C, high‐density lipoprotein cholesterol; LDL‐C, low‐density lipoprotein cholesterol; MRE, magnetic resonance elastography; MRI‐PDFF, magnetic resonance imaging‐proton density fat fraction; TG, triglyceride.

### Changes in factors associated with changes in MAST score

**Figure 2:**
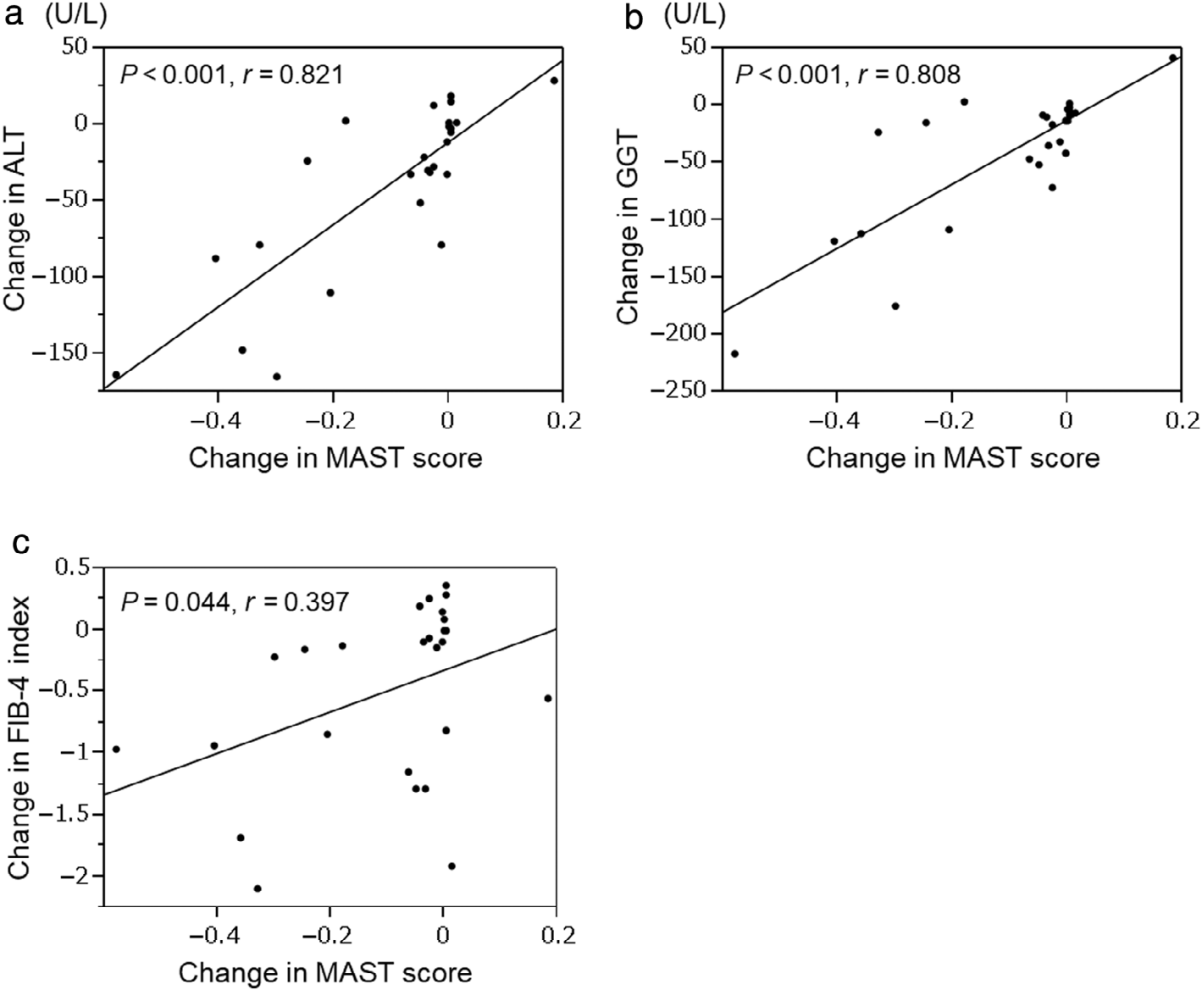
Correlations between changes in magnetic resonance imaging‐aspartate aminotransferase (MAST) score and alterations in the levels of (a) alanine aminotransferase (ALT), (b) gamma‐glutamyl transferase (GGT), and (c) fibrosis‐4 (FIB‐4) index during pemafibrate treatment.

Table 5 presents the changes in ALT and GGT levels and the FIB‐4 index, which show positive correlation with changes in the MAST score (*r* = 0.821, *P* < 0.001; *r* = 0.808, *P* < 0.001; and *r* = 0.397, *P* = 0.044, respectively). The level of HDL‐C also showed a positive correlation with changes in the MAST score (*r* = 0.418, *P* = 0.034). Overall, no significant correlation was observed between changes in the MAST score and changes in other parameters, including low‐density lipoprotein‐cholesterol (LDL‐C), TG, and non‐HDL‐C. The scatter plots in Figure 2 show the significant correlation between changes in the MAST score and changes in the levels of ALT, GGT, and FIB‐4 index after 48 weeks of pemafibrate treatment.

**Table 5:** Association between changes in magnetic resonance imaging‐aspartate aminotransferase (MAST) score and changes in factors

Change in	Change in MAST score	
	Correlation coefficient	*P*‐value
Total bilirubin (mg/dL)	−0.037	0.874
AST (U/L)	0.859	<0.001
ALT (U/L)	0.821	<0.001
GGT (U/L)	0.808	<0.001
eGFR (mL/min/1.73 m^2^)	0.099	0.631
Triglyceride (mg/dL)	−0.067	0.747
LDL cholesterol (mg/dL)	0.125	0.544
HDL cholesterol (mg/dL)	0.418	0.034
Non‐HDL cholesterol (mg/dL)	0.190	0.352
Platelets (10^4^/μL)	0.262	0.196
FIB‐4 index	0.397	0.044

ALT, alanine aminotransferase; AST, aspartate aminotransferase; eGFR, estimated glomerular filtration rate; FIB‐4, fibrosis‐4; GGT, gamma‐glutamyl transferase; HDL‐C, high‐density lipoprotein cholesterol; LDL‐C, low‐density lipoprotein cholesterol; TG, triglyceride.

## Discussion

According to the present study, pemafibrate led to improvements in the evaluation of significant fibrosis using the rule‐out and rule‐in criteria based on the MEFIB index and the MAST score. The change in the MAST score showed a correlation with improvements in liver enzymes such as ALT and GGT. To the best of our knowledge, this is the first report focusing on the potential effects of pemafibrate using the MEFIB index and MAST score.

Pemafibrate treatment significantly reduced the AST, ALT, and GGT levels. Additionally, a significant reduction in TG and non‐HDL‐C and an increase in HDL‐C were observed following pemafibrate treatment. These results are consistent with those of previous reports.^33^, ^34^ Recent studies have reported a significant decrease in the FAST score following pemafibrate treatment^16^, ^19^, ^35^ and its correlation with the changes in ALT^16^ or GGT levels.^35^ In this study, we found a significant reduction in the MAST score following pemafibrate treatment and a correlation between the reduction of the MAST score and the changes in ALT and GGT levels. The reduction of ALT levels indicates an improvement in histological inflammation in patients with biopsy‐proven NASH.^36^ The correlation between the reduction of the MAST score and changes in the ALT level confirmed that pemafibrate treatment prevents disease progression in MASLD by exerting anti‐inflammatory effects, as suggested by Hatanaka *et al*.^16^

However, changes in the levels of serum lipids, such as TG, HDL‐C, and non‐HDL‐C, did not show any correlation with changes in the MAST score. Pemafibrate treatment significantly improved LSM on MRE, but did not reduce the liver fat content measured using MRI‐PDFF, as previously reported by Nakajima *et al*.^17^ Although the mechanism underlying the effect of pemafibrate on liver stiffness and hepatic steatosis requires further analysis, in STAM NASH model mice, pemafibrate led to enhanced accumulation of F4/80‐positive macrophages, ballooning degeneration of hepatocytes, and NAFLD activity score, without reducing the total liver fat content.^37^ Pemafibrate induces an increased number and reduced size of lipid droplets, resulting in reduced hepatic macrovesicular steatosis and decreased expression of inflammation‐and fibrosis‐related genes.^37^ This study using the NASH mice model confirmed the findings of Nakajima *et al*.^17^

Tamaki *et al*. reported that the area under the receiver operating characteristics curve (AUROC) (95% CI) of the MEFIB index (0.860 [0.81–0.91]) was significantly higher than that of the FAST score (0.757 [0.69–0.82]) in diagnosing significant fibrosis (≥stage 2). When used as the rule‐in criteria by the MEFIB index, the positive predictive value (PPV) for significant fibrosis was 91.2–96.0% for MEFIB and 74.2–89.2% for FAST. When used as the rule‐out criteria by the MEFIB index, the negative predictive value (NPV) of significant fibrosis was 85.6–92.8% for MEFIB index and 75.8–88.3% for FAST.^27^ Similarly, the MAST score showed high performance and discrimination in the validation cohort for identifying Fibro‐NASH (AUROC 0.93 [0.88–0.97]. In the validation cohort, a 90% specificity cut‐off of 0.242 corresponded to a sensitivity of 75.0%, PPV of 50.0%, and NPV of 96.5%, whereas a 90% sensitivity cut‐off of 0.165 corresponded to a specificity of 72.2%, PPV of 29.4%, and NPV of 98.1%. Compared to the FAST score, the MAST score showed a higher AUROC and an overall better discrimination.^25^ Moreover, compared to previous noninvasive tests, the MEFIB index and the MAST score demonstrated greater accuracy in the identification of patients at high risk of liver disease progression. In our study, using the MEFIB index and the MAST score, we showed that pemafibrate treatment led to significant improvements in the evaluation of significant fibrosis. Ajmera *et al*. proposed that LSM on MRE and the MEFIB index are associated with liver‐related events, including the incidence of hepatocellular carcinoma and hepatic decompensation.^29^ Consequently, our study indicated that pemafibrate treatment has the potential to reduce liver‐related events in patients with MASLD. In addition, a weak positive correlation was observed between the changes in the MAST score and alterations in HDL‐C levels during pemafibrate treatment. As vitamin E, an antioxidant, can improve both ALT levels and histological activity, oxidative stress has been associated with NAFLD progression.^38^ A recent report showed that the antioxidant capacity of HDL‐C is impaired in NAFLD.^39^ The elevation of HDL‐C levels induced by pemafibrate treatment might not be associated with MASLD improvement, potentially due to dysfunctional HDL‐C.

We demonstrated that higher levels of AST, ALT, and GGT, as well as higher scores of FIB‐4 index, LSM on MRE, and MAST at baseline, were associated with the effects of pemafibrate treatment. Considering these baseline factors, our findings suggested that the effect of pemafibrate on patients with MASLD and hypertriglyceridemia was associated with liver inflammation and fibrosis at baseline. Pemafibrate may be advantageous for treating patients who exhibit increased liver inflammation and fibrosis. However, further research is required to assess the histological improvements, particularly in patients with advanced liver fibrosis.

Our study had some limitations. This was a single‐center retrospective observational study, which might have introduced selection bias. Another limitation is the absence of a control group in our study design. Although several Phase IIb and Phase III trials have already incorporated noninvasive tests such as the MAST and FAST scores to screen for patients with “at risk” NASH, histopathological evaluation of the liver was not conducted as part of these trials. Although noninvasive tools, including scoring systems and quantitative imaging assessments, have demonstrated strong correlations with liver biopsy results, the responses of the MEFIB index and MAST score to treatment were not confirmed through histological examination. Additionally, the small sample size may have affected our ability to identify the factors associated with the effects of pemafibrate treatment.

## Conclusion

In conclusion, our findings show that pemafibrate led to improvements in the MEFIB index and MAST score, as well as liver function, thereby reflecting its hepatic anti‐inflammatory effects. Pemafibrate is a promising therapeutic agent for preventing MASLD progression complicated by hypertriglyceridemia.

## Ethics statement

This study has been approved by the Ethical Committee of Nippon Koukan Hospital.

## Patient consent

This study is retrospective study. Patients were not required to give informed consent to the study because the analysis used anonymous clinical data that were obtained after each patient agreed to the treatment. Also, we applied the opt‐out method to obtain consent for this study by using a poster (described later). The poster was approved by the Ethical Committee of Nippon Koukan Hospital.

## Data Availability

The data used in the present study might be available from the corresponding author upon reasonable request.
